# Peri-implant conditions and marginal bone loss around cemented and screw-retained single implant crowns in posterior regions: A retrospective cohort study with up to 4 years follow-up

**DOI:** 10.1371/journal.pone.0191717

**Published:** 2018-02-05

**Authors:** Jun-Yu Shi, Jie-Ni, Long-fei Zhuang, Xiao-Meng Zhang, Lin-Feng Fan, Hong-Chang Lai

**Affiliations:** 1 Department of Dental Implantation, Shanghai Ninth People’s Hospital, Shanghai Key Laboratory Stomatology, Shanghai Jiaotong University, School of Medicine, Shanghai, China; 2 Shanghai Adore Dental Care, Shanghai, China; 3 Department of Radiology, Shanghai Ninth People’s Hospital, Shanghai Key Laboratory Stomatology, Shanghai Jiaotong University, School of Medicine, Shanghai, China; ITALY

## Abstract

The aim of the present study was to identify the peri-implant conditions (bleeding on probing (BOP), pocket probing depth (PPD), modified plaque index (mPI)) and marginal bone loss (MBL, marginal bone level change between follow-up and occlusal loading) around cemented and screw-retained posterior single crowns on tissue-level implants. The study was a retrospective cohort study with up to 4 years (mean 2.5 years) follow-up. Patients with either cemented or screw-retained crowns in posterior regions were included. Implant survival, technical complications, BOP, PPD, mPI, MBL, biologic complications (peri-implant mocositis and peri-implantitis) were evaluated. Mann-Whitney U test was used to test the difference between the screw-retained group (SG) and cemented group (CG). 176 patients (SG: 94, CG: 82) were included. The implant survival rates were 100% in SG and 98.8% in CG. Prosthetic screw loosening was found in 8 restorations (8.7%) at follow-up visit. Peri-implant mucositis rate was significantly higher in the SG group (42.1%) than that in the CG group (32.2%) (P = 0.04). Six patients (6.38%) in the screw-retained group and 5 patients (6.10%) in the cemented group were diagnosed with peri-implantitis, the difference did not reach statistical significance (P>0.05). No significant difference of PPD, mPI and MBL were found between two groups (P = 0.11, 0.13 and 0.08, respectively). High implant survival rates were achieved in both groups. Cemented single crowns on tissue-level implants showed comparable peri-implant conditions in comparison with two-piece screw-retained crowns. Well-designed prospective cohort or randomized controlled clinical trials with longer follow-up are needed to confirm the result.

## Introduction

Dental implants have become a routine treatment strategy in partial and edentulous patients[1]. One of the important clinical decisions in implant treatment was the choice of implant/abutment connection type connecting restorations and implant abutments: cemented or screw-retained prostheses? Numerous studies evaluated the clinical outcomes of cemented and screw-retained prostheses, however, only limited conclusive evidences were available, which could help the clinicians to make the clinical decision[2, 3].

Screw-retained prostheses were first introduced for full-arch implant-supported prostheses in edentulous patients[4]. Subsequently, cemented prostheses were also widely applied due to the ease of restorability during the 2000’s [5]. Both screw-retained and cemented retention have been used in connection of implant-supported single crowns, fixed partial denture and full arch prostheses.

Previously, cemented prostheses were considered to be accompanied by higher biologic complication rates, while screw-retained prostheses were accompanied by higher technical complication rates[6, 7]. Larger marginal micro-gaps were found around cemented prostheses than those around screw-retained prostheses, which could lead to more biofilm accumulation and higher prevalence of peri-implant infections (for review, see in [8]. In addition, it was reported that residual cement might lead to peri-implant infection, especially when cement margins were relatively deep[9].

However, with the development of CAD/CAM technology, the precision of implant-supported restorations was significantly improved. A recent review showed that it was possible to obtain a marginal micro-gap less than 80μm with milled CAD/CAM restorations[10]. In addition, the specific design of implants, such as tissue-level implants with smooth implant necks, could make cement margins more coronal, thus reducing the risk of peri-implant inflammation caused by residual cement[11].

On the other hand, the rates of prosthetic screw loosening were relatively high (5-year rates: 3.6%-10.8%)[12]. Screw loosening would lead to the micro-movement of the restorations, enlargement of marginal micro-gaps and peri-implant inflammation [13].

Nowadays, tissue-level implants are still widely used in daily clinical practice. Unfortunately, only limited evidences regarding biologic complications of cemented and screw-retained prostheses on tissue-level implants are available now.

Thus, the aim of the present study was to evaluate the peri-implant conditions and marginal bone loss around cemented and screw-retained single crowns on tissue-level implants in posterior region. The null hypothesis was no significant difference regarding biological complications will be found between cemented and screw-retained restorations.

## Materials and methods

### Study design and patient data

The study was a retrospective cohort study with up to 4 years follow-up (mean 2.5 years). Patients who received implant therapy in the Department of Oral and Maxillofacial Implants, Shanghai Ninth People’s Hospital, from January 2012 to May 2015, were recruited. Their medical data were reviewed. Eligible patients were invited for a follow-up visit. All participants signed informed consent before they were included in the present study. The study protocol and informed consent were approved by the Ethics Committees of Shanghai Ninth People’s Hospital (Hu-Lun 2016_215, Shanghai, China). In addition, all procedures were conducted in full accordance with the World Medical Association Declaration of Helsinki (version, 2002).

### Inclusion criteria:

1. Patients with single implant crowns in posterior region (distal to canine tooth);
2. Patients with periodontal treatment before implant surgery (Full Mouth Plaque Score <20%, Full Mouth Bleeding Score<20%, pocket probing depth<5mm);
3. Patients with either cemented or screw-retained restorations;
4. Patients with natural teeth adjacent to single implant crowns (mesial);
5. Patients with a minimal follow-up of 12 months.

### Exclusion criteria:

1. Patients with bone augmentation procedures;
2. Patients with uncontrolled periodontal diseases at baseline (Residual pocket depth>5mm, Full mouth bleeding score ≤20%, Full mouth plaque score ≤20%);
3. Uncontrolled diabetes mellitus (Fasting blood-glucose>7.2mmol/L, Glycosylated hemoglobin >7%);
4. Heavy smokers (>10 cigarettes/day);
5. Patients with parafunction (i.e. bruxism);
6. Unwilling to participate in the present study.

### Screening

The patients included in the study were divided into two groups according to the implant-abutment connection type: cemented group (CG) and screw-retained group (SG). All implants in the present study were Straumann® Standard (2.8mm polished neck) or Standard Plus (1.8mm polished neck) SLA implants (Institut Straumann AG, Basel, Switzerland). Appropriate implants were selected based on mucosal thickness to make the implant shoulders 0.5-1mm below the mucosal margins. Peri-apical radiographs with paralleling technique (XCP Instruments, Rinn Corporation Elgin, Elgin, IL, USA) were performed before prosthetic treatment (three months after implant surgery). For patients who received submerged healing protocol, a second-stage surgery was performed. Two weeks later, impressions were performed. Y-TZP frameworks (Lava Zirconia, 3M ESPE) were fabricated with CAD/CAM technique in the lab. Then, the frameworks were veneered with ceramic (VM9, VITA). For cemented crowns, standardized titanium abutments (synOcta® 048.606, 048.608 and 048.609 were adopted. The cement used in the present study was HY-bond Glass-ionomerCement (CX, Shofu INC, Tokyo, Japan). For screw-retained crowns, two-piece protocol and titanium abutments (synOcta® 048.601) were adopted. 35Ncm was applied to the abutment screw (connecting the implant and abutment) and 15Ncm was applied to the prosthetic screw (connecting the abutment and restoration). If more than one restoration in one patient met the inclusion criteria, one restoration was randomly selected. The following parameters were recorded: age, gender, history of periodontitis, implant location, implant length, implant diameter and follow-up.

### Outcome assessment

#### Survival and technical complications

Survival rate was defined by percentage of single crowns which remained and never been replaced. In addition, technical complications, such as veneer chipping, abutment or screw loosening and fracture of implant, were recorded.

#### Peri-implant conditions

The following parameters were recorded at follow-up visits: bleeding on probing + % (BOP+%) modified plaque index (mPI), pocket probing depth (PPD) and suppuration. The peri-implant examination was performed by a well-trained periodontist (N.J.) with PQW Williams probe (Hu-Friedy, Chicago Ill, USA). The probe was inserted parallel to the implant surface and directed apically toward the perceived location of the apex until slight resistance was felt. BOP% and PPD were recorded. A score was given to four areas of the implant restoration. The final result was calculated as the mean value of the four scores. Modified plaque index of each implant site (Mombelli et al. 1987) was also recorded at each follow-up visit.

#### Marginal bone loss

Digital peri-apical and panoramic radiographs were taken at baseline and follow-up visits. A paralleling technique (XCP Instruments, Rinn Corporation Elgin, Elgin, IL, USA) was used. Radiographic analysis was conducted by a software program (SIDEXIS 1.12, Sirona Dental System GmbH, Bensheim, Germany). The examinations were performed by two calibrated dentists (Z.X.M & F.L.F). The implant length was used as reference for calibration. The distance from restoration margin to the most coronal level of implant-bone contact at the mesial and distal sites was recorded. The final result was calculated as the mean value of the two sites. The alteration of the distance between baseline and follow-up visit was defined as marginal bone loss.

#### Biological complications

Peri-implantitis was chosen as the primary endpoint, which was defined as advanced marginal bone loss (>2mm) combined with BOP+ and mucosal suppuration. Peri-implant mucositis, which was defined as BOP+ and mucosal suppuration without advanced marginal bone loss, was also recorded.

### Data analysis

Mean and standard deviation were calculated for quantitative variables. The inter-examiner reliability was determined by intra-class correlation coefficient (ICC) for marginal bone loss. The skewness and kurtosis test was used to test for normality of distribution of the data. Mann-Whitney U test was used to test the difference of clinical parameters and marginal bone loss between two groups. Chi-Square test was used to test the difference of age, gender, implant location, implant diameter, implant length, follow-up, history of periodontitis and peri-implantitis rates between two groups. The level of significance was set at α = 0.05. Data analysis was performed using a statistical software package STATA (version 11.0; StataCorp, College Station, TX, USA).

## Results

### Survival and technical complications

One hundred and ninety two patients fulfilled the inclusion criteria, 176 patients (SG: 94, CG: 82) were included. Sixteen patients (8.33%) refused to participate in the follow-up examination. Four patients changed their contact information, 11 patients moved out of the city and 1 patient passed away. Table 1 shows the characteristic of included patients. No significant difference of age, gender, implant location, implant diameter, implant length, follow-up, history of periodontitis, opposing occlusion and healing protocol were found between two groups. All included patients were with natural teeth adjacent to single implant crowns in the mesial surface. 22 patients (9 in SG and 13 in CG) lost their adjacent tooth in the distal surface, while other 154 patients were with natural teeth in the distal surface.

**Table 1:** Summary of patient characteristics.

	SG (n = 94)	CG (n = 82)	P value
**Mean age (years)**	49.6(27–72)	46.8 (28–70)	0.06
**Male/female ratio**	38/56	34/48	0.93
**Implant location: Maxillae/Mandible**	43/51	42/40	0.67
**Implant diameter: 4.1/4.8mm**	63/31	58/24	0.82
**Implant length: 8/10mm**	26/68	27/55	0.58
**Follow-up (months)**	30.2(12–51)	31.4(12–52)	0.25
**History of periodontitis: Y/N**	28/66	31/51	0.43
**Opposing occlusion: T/F/R**	60/15/19	55/21/6	0.02*
**1-stage/2-stage**	86/8	75/7	0.99

*, P<0.05.

T, natural teeth; F, fixed dental prostheses; R, removal dental prostheses.

**Fig 1:**
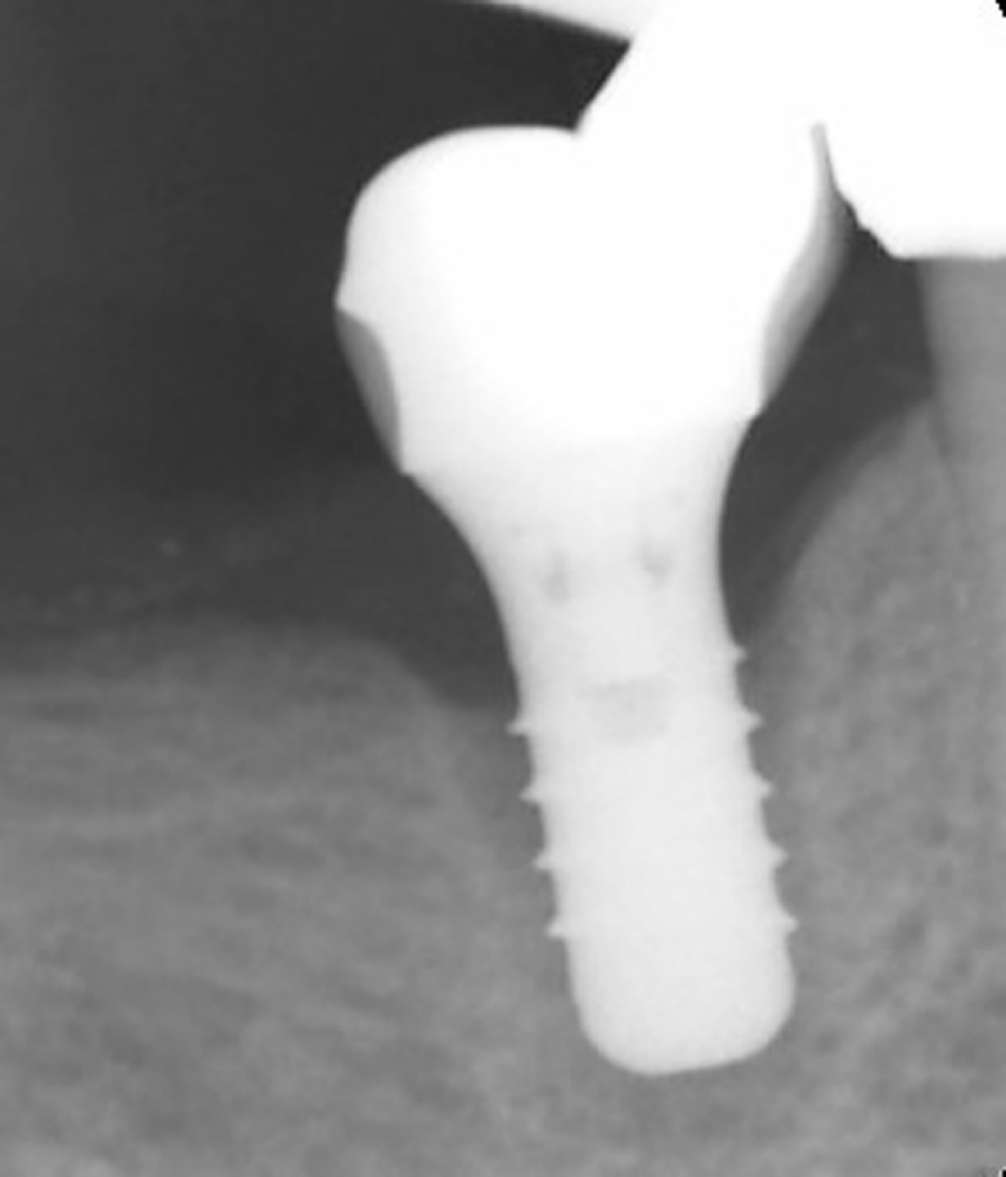
Radiograph that identified a failed implant (3.2 years after implant surgery).

**Fig 2:**
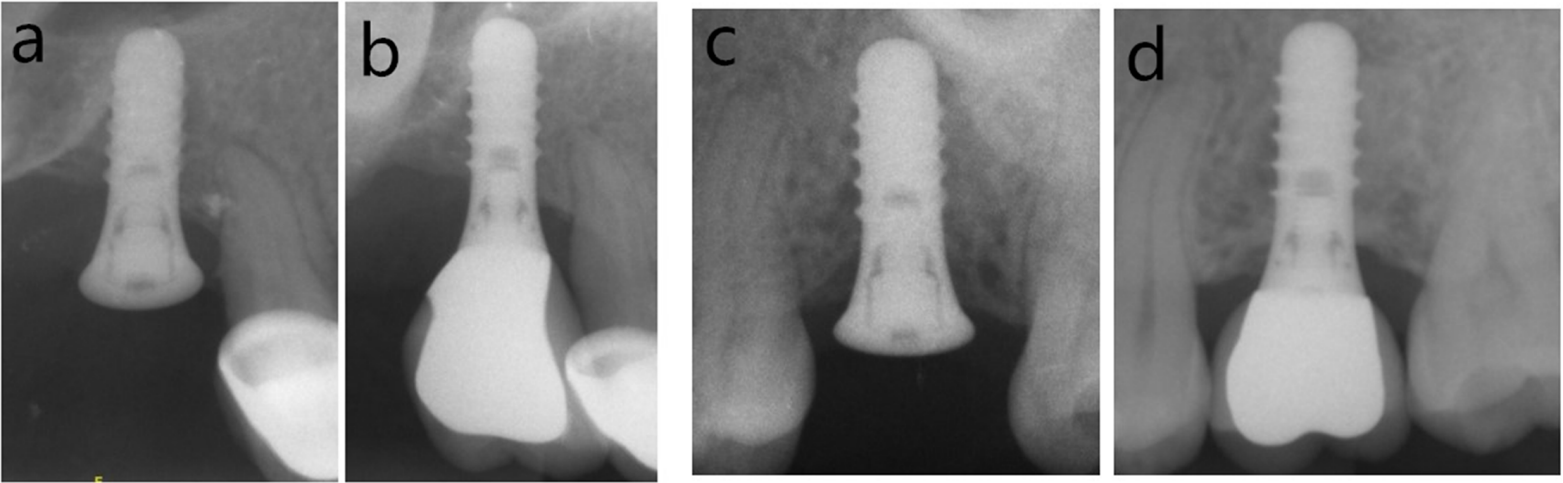
Radiographs in SG group: a) baseline, b) follow-up, and in CG group: c) baseline, d) follow-up.

**Fig 3:**
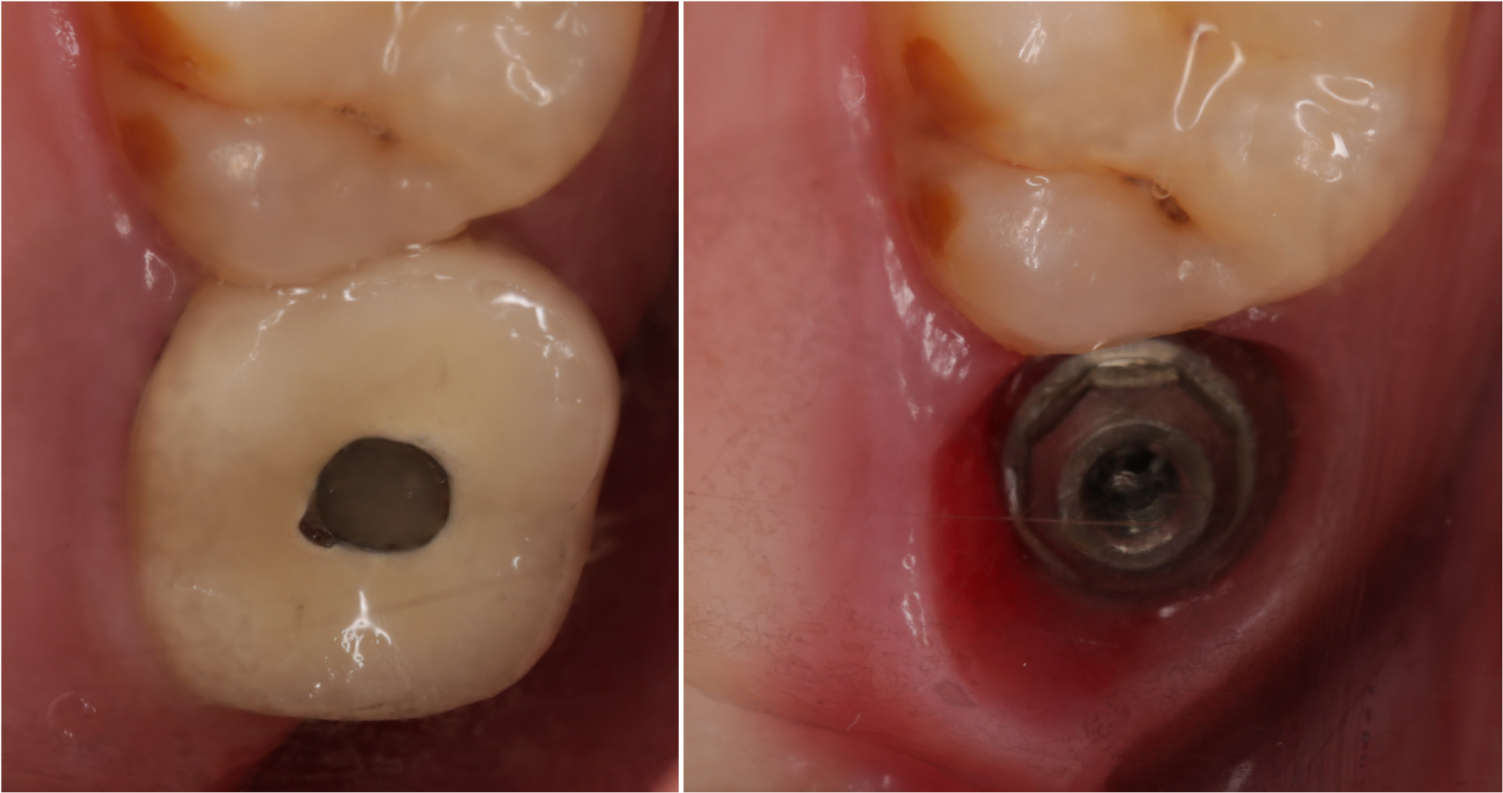
Occlusal view of a crown with a loose abutment screw and swollen mucosa around implant platform.

During the observation period, one implant loss was found in cemented groups due to peri-implant infection. Fig 1 showed the radiograph that identified a failed implant (3.2 years after implant surgery). Thus, the implant survival rate was 100% in SG and 98.8% in CG. Veneer chipping was found in 6 restorations in SG (6.4%) and 8 restorations in CG group (9.8%) at follow-up visit. Fig 2 showed the radiographs in SG and CG groups at baseline and follow-up. Prosthetic screw loosening was found in 8 restorations (8.7%) at follow-up visit. No other technical complications were found. Fig 3 showed the occlusal view of a crown with screw loosening and swelling mucosa around implant platform.

### Clinical and radiological parameters

**Fig 4:**
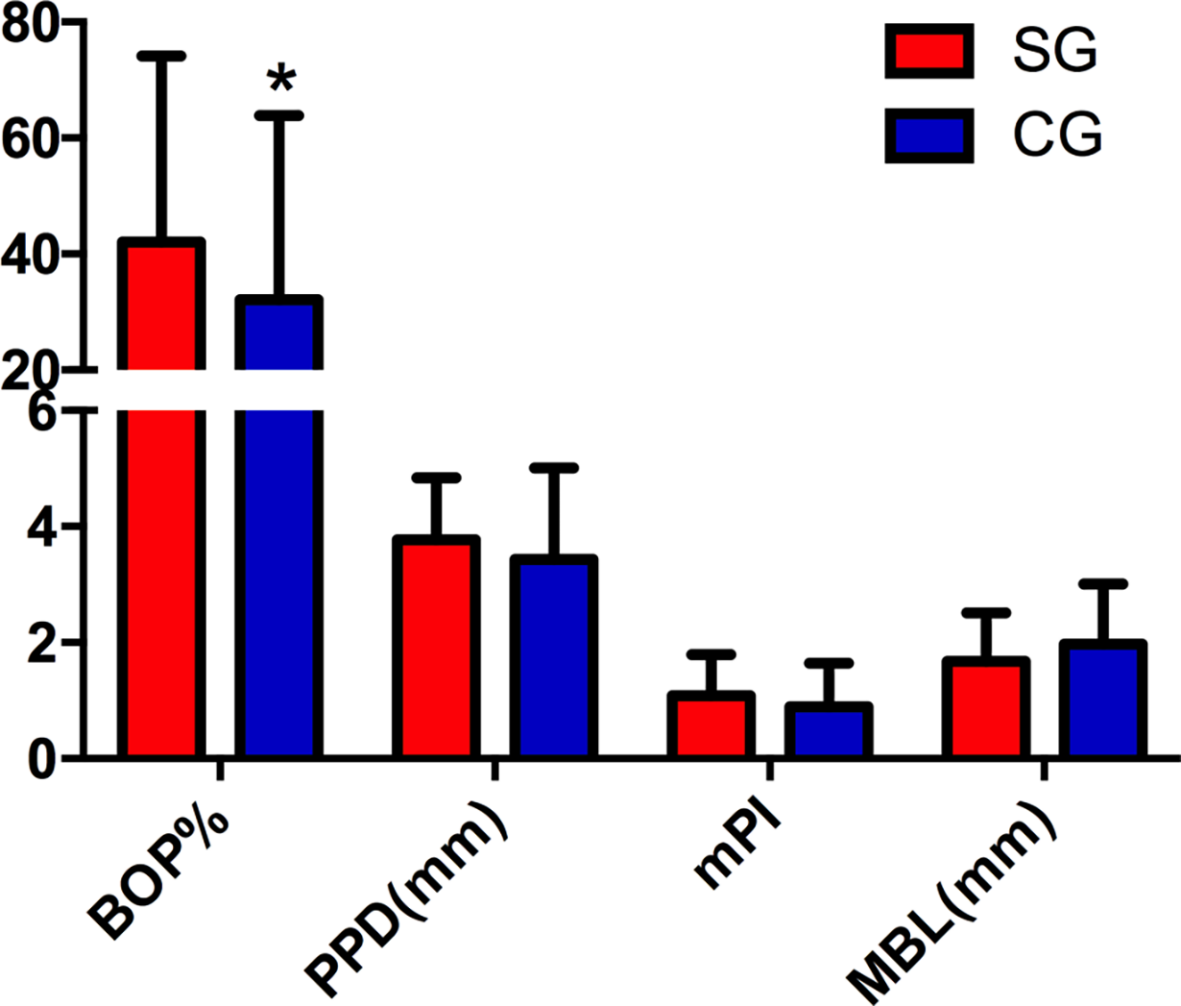
Comparison of clinical and radiological parameters between cemented and screw-retained crowns, *, significant difference between two groups.

Table 2 shows the peri-implant conditions and marginal bone loss of included restorations. BOP% was 42.1% and 32.2% in SG and CG groups, respectively. No significant difference of PPD, mPI and MBL were found between two groups (P = 0.11, 0.13 and 0.08, respectively). Fig 4 showed the comparison of the clinical and radiological parameters between two groups.

**Table 2:** Clinical and radiological parameters.

	SG (n = 94)	CG (n = 82)	P value
BOP% (mean)	42.10	32.16	0.04*
Median (range)	50 (0–100)	25 (0–100)	
PPD(mm) (mean)	3.78	3.43	0.11
Median (range)	3.5 (1.5–6.7)	3.3 (1.5–8.3)	
mPI (mean)	1.09	0.89	0.13
Median (range)	1 (0–2)	1 (0–2)	
Marginal bone loss (mean)	1.67	1.97	0.08
Median (range)	1.49 (0.58–7.25)	2.11 (0.38–6.54)	

BOP%,bleeding on probing%; PPD, pocket probing depth; mPI, modified plaque index; SG, screw-retained group; CG, cemented group

* P<0.05.

### Biological complications

Peri-implant mucositis rate was significantly higher in SG group (42.1%) than that in CG group (32.2%) (P = 0.04). Six patients (6.38%) in screw-retained group and 5 patients (6.10%) in cemented group were diagnosed as peri-implantitis, the difference did not reach statistically significant (P>0.05). They received surgical mechanical debridement and laser therapy.

## Discussion

The peri-implant condition and marginal bone loss around cemented and screw-retained single implant crowns were evaluated in the present study. Strict inclusion criteria were adopted to avoid confounding factors. All patients received oral hygiene instruction and periodontal treatment before implant surgery. In addition, heavy smokers, patients with uncontrolled periodontitis, uncontrolled diabetes mellitus, para-function and augmentation procedures were excluded.

The post hoc power analysis was performed based on the previous systematic review results (biological complications in SG and CG: 34.3% and 14.4%, respectively) [14] using software (N-solution 2008 Build Version 2.000). The result showed the 70 implant crowns in each group and 140 implant crowns in total could achieve 80% power when the level of significance was set at 0.05. Thus, the sample size of the present study was enough to explore the clinical differences between two groups.

Several studies claimed that cemented restorations could lead to significantly higher biologic complications and peri-implant infections, though the implant survival seemed not to be affected [2, 6, 15–17]. In this study, similar peri-implantitis rate between SG and CG was found. However, significantly higher peri-implant mucositis rate was found in SG group than that in CG group (P<0.01). This result was partially in line with a recent systematic review[14]. It was reported that the estimated biologic complication rates per 100-year of cemented restorations (7.01%) were slightly lower than that of two-piece screw-retained restorations (10.51%). It is worth mentioning that the prosthetic screw loosening rate was 8.7% in the present study, which was slightly higher than the event rates reported in the literature (annual rates: 2.29%)[12]. According to the manufacture’s instruction, only 15Ncm was applied on the prosthetic screw. This might be the weakness of the restoration and lead to the high loosening rate. The micro-movement of the restorations and enlargement of marginal micro-gap caused by prosthetic screw loosening might be the possible reason for higher BOP positive sites of screw-retained crowns in the present study[18].

In this study, only HY-bond Glass-ionomer Cement (CX, Shofu INC, Tokyo, Japan) was used and low peri-implantitis rates (6.10%) were found. On the other hand, Linkevicious reported contradictory results in a similar study with resin cement[19]. In addition, Kotsakis suggested that the difference in results between various studies was due to use of different cements[20]. This indicated that Glass-ionomer cement might reduce the risk of peri-implantitis.

It is believed that biologic width around one-piece tissue-level implants was more similar to natural teeth than that around two-piece bone-level implants [21, 22]. In addition, tissue-level implants with a smooth implant neck (1.8mm or 2.8mm)can make the cement margin more coronal. A previous prospective cohort study have reported significantly geater amount of undected cement is found when the margin position is deeper[11]. Better soft-tissue concealing and relatively shallow cement margin of tissue-level implants could be the possible reason for lower BOP positive sites of cemented crowns in the present study.

In this study, the percentage of patients with history of periodontitis in the SG group (37.8%) was slightly higher than that in the CG group (30.4%), but the difference did not reach statistical significance (P = 0.43). It was demonstrated that a history of periodontitis was a risk factor for peri-implant diseases[23]. This might be the possible confounding factor for interpreting the result. More well-designed prospective cohort or randomized controlled clinical trials are needed to confirm the result.

No significant difference of modified plaque index and pocket probing depth was found between two groups. This indicated that both cemented and screw-retained crowns were cleanable. And the oral hygiene was comparable in the two groups. Unfortunately, comparison of pocket probing depth changing from baseline to follow-up between two retention types was impossible due to the lack of baseline pocket probing depth. Future studies should establish the baseline of pocket probing depth and evaluate the pocket probing depth changing between two groups.

No significant difference of marginal bone loss was found between two groups. This result was in line with a previous meta-analysis[24]. Overall, 9 studies were included. Pooled mean marginal bone loss was 0.53mm (95%CI: 0.31–0.76mm) for cemented restorations and 0.89mm (95%CI: 0.45–1.33mm) for screw-retained restorations. No evidence is available to support differences of marginal bone loss between two retention types. This indicated that the retention type would not affect the marginal bone loss of tissue level implant crowns.

The present study showed several shortcomings. Firstly, the selection bias was relatively high due to the retrospective design, though strict inclusion criteria were adopted to avoid confounding factors. In addition, the lack of baseline of pocket probing depth made the assessment of pocket probing depth changing between two groups impossible. Last but not least, the external validity was relatively poor due to the strict inclusion criteria. Cautions must be taken to extrapolate this result to other implant-supported restorations.

## Conclusion

With the previously mentioned limitations, high implant survival rates were achieved in both groups. Cemented single crowns on tissue-level implants showed comparable peri-implant conditions when compared with two-piece screw-retained crowns. Well-designed prospective cohort or randomized controlled clinical trials with longer follow-up are needed to confirm the result.

## Supporting information

S1 Table STROBE checklist. (DOC)
